# Research trends in college students' sleep from 2012 to 2021: A bibliometric analysis

**DOI:** 10.3389/fpsyt.2022.1005459

**Published:** 2022-09-20

**Authors:** Jingxin Zhou, Junchao Qu, Siqi Ji, Yuan Bu, Yicheng Hu, Huiping Sun, Mengxin Xue, Ting Zhou, Jiling Qu, Yongbing Liu

**Affiliations:** School of Nursing and Public Health, Yangzhou University, Yangzhou, China

**Keywords:** college students, sleep, CiteSpace, bibliometric analysis, research hotspots

## Abstract

**Background:**

A great proportion of college students experience various sleep problems, which damage their health and study performance. College students' sleep problems, which are caused by several factors, have been easily ignored before. In the past decade, more research has been published to expand our understanding of undergraduates' sleep. The purpose of the study is to explore the research hotspots and frontiers regarding college students' sleep using CiteSpace5.8.R3 and offer guidance for future study.

**Methods:**

We retrieved relevant literature from the Web of Science Core Collection Database and imputed the downloaded files into CiteSpace5.8.R3 for visualization analysis. We generated network maps of the collaborations between authors, countries, institutions, the cited journals, and co-occurrence keywords. The analysis of keywords clusters, timeline views, and keywords citation bursts help us identify the hotspots and research trends.

**Results:**

A total of 1,841 articles related to college students' sleep, published from 2012 to 2021, were selected. The number of publications gradually increased. Karl Peltzer was the most prolific authors with 15 publications. The United States and Harvard University separately contributed 680 and 40 articles and had the greatest impact in this field. SLEEP ranked first in the frequency of cited journals. The article published by Lund HG was the most influential publication. Based on the analysis of keywords, we summarized research hotspots as follows: current status, affecting factors, and adverse outcomes of college students' sleep. The frontiers were the further understanding of the relationships between sleep and mental and physical health, and various interventions for sleep disorders.

**Conclusion:**

Our study illustrates the research hotspots and trends and calls for more research to expand the findings. In the future, the cooperation between institutions and authors needs to be strengthened. The complex relationships between sleep and mental and physical health and problematic substance use disorders are necessary to be explored. Longitudinal studies or randomized controlled trials should be constructed to verify the current findings or assumptions.

## Introduction

Sleep is considered as a quickly reversible condition of reduced responsiveness, reduced motor activity, and reduced metabolism (1). It is regulated by circadian, homeostatic, and neurohormonal processes (2). It is generally agreed that sleep can aid memory consolidation (3), which is beneficial to people's ability to learn knowledge and skills.

College students experience changes in lifestyle and face stress stemming from study and work, which tend to lead to various sleep disorders. A study showed that 40–60% of American college students had sleep problems and a third had a shortened sleep duration (4). Another study conducted in Luxembourg and Germany found that a third of students suffered from daytime sleepiness (5). The percentages of Chinese students with sleep complaints and suffering from insomnia were 20.3 and 23.6%, respectively (6). Poor sleep quality has been associated with a series of negative consequences, such as obesity, depression, cardiovascular disease, and all-cause mortality (7–9). College students were thought to be in the best functional state, so their sleep disorders were likely neglected in the past.

Fortunately, scholars have gradually become more aware of the importance of sleep and increasing numbers of studies have been conducted on the topic. The prevalence, diverse affecting factors, and adverse outcomes of sleep problems have been explored by scholars (6, 10–12). In recent years, different kinds of interventions aimed to solve sleep problems also have been studied further (13, 14). Although some qualitative reviews summarized the characteristics of college students' sleep, bibliometric methods have not been used to comprehensively analyze the distribution and contribution of research in this field up to now (15, 16). Hosseini et al. and Darko et al. pointed out that the qualitative review may have the potential bias of subjectivity (17, 18). Therefore, it is necessary to systematically review college students' sleep with quantitative methods.

Bibliometric analysis refers to mathematical and statistical scientific workflows, which helps present the knowledge structure of a research domain (19). It includes three main analyses: a co-authorship analysis, a co-word analysis, and a co-citation analysis (20). Scholars can quickly clarify the characteristics of literature, the evolution process, and research hotspots by bibliometrics. A study showed that the literature production trend using bibliometric analysis in medical research was active, and more than one-third of bibliometric articles indexed in Scopus were from the medical field (21). At present, scholars have used bibliometric analysis to study treatments for insomnia and different populations' sleep disorders (22–24). But few research focused on healthy people, even on college students.

CitsSpace5.8.R3 is a widely used visualization application in many fields, such as environmental science, pedagogy, forestry, communication, public health, and tourism (25, 26). It is accessible for free and can process literature data. Therefore, we used CiteSpace5.8.R3 as our visualization analysis tool and selected articles about college students' sleep from the Web of Science Core Collection Database. We analyzed the literature from 2012 to 2021 as a result that it would reveal more latest trends emerged in recent years. By means of CitesSpace5.8.R3, we can generate vivid network maps of co-authors, countries, and institutions; co-cited references; co-occurring keywords and clusters; and a keyword burst.

Our research is designed to solve the following problems: (a) What is the distribution of literature on college students' sleep in the past decade? (b) Who are the important contributors on college students' sleep? (c) What are the research hot spots and frontiers in this field? Our purpose is to use these generated maps or tables to objectively understand the current situation, research hot spots, and the research frontiers of college students' sleep. To the best of our knowledge, this is the first study to quantitatively analyze college students' sleep using bibliometric tools, which can complement the lack of quantitative literature review in this field. Additionally, our research will help interested scholars extract important information to support their current research. It will also make it easier for them to understand the development of this field from a scientific perspective and provide a reference for further clinical work and sleep hygiene practice.

The remainder of the paper was arranged as follows. In the Data and Method section, we elaborated on the data collection procedures, the analysis tool, and the analysis process. The Results and Analysis section showed and explained a series of generated knowledge graphs and tables. In the Discussion section, we summarized the current research hotspots, development trends, and research frontiers in this field through a comprehensive analysis of all the results. The advantages and disadvantages of our research and the future research directions were pointed out in the end.

## Data and method

### Data collection

The data for this review were collected on the Web of Science on July 22, 2022. It is acknowledged that Web of Science has a considerable influence on the world. We chose the Web of Science Core Collection as the database. The indexes used were the Science Citation Index Expanded and the Social Science Citation Index of the Web of Science. The first article on college students' sleep on the Web of Science was published in 1999 and since then the number of papers has increased. To learn about the latest development trend in this field, we chose to retrieve articles from 2012 to 2021. The search strategies were as follows: TS = (“college student^*^” OR “university student^*^” OR “undergraduate^*^” OR “undergraduate student^*^”) AND (“sleep”). The document type was article or review with a timespan ranging from January 1, 2012 to December 31, 2021. The language selected was English because it is an international language and many articles were published in English. The exclusion criteria were: (1) literature types of conference papers, newspapers, notes, and other non-academic reports; (2) duplicate articles; (3) unrelated articles. The search queries were listed in Table 1. A total of 1,841 records were obtained. The data were downloaded in plain text format with full records and references and were then imported into CiteSpace5.8.R3 for further analysis.

**Table 1:** The search queries.

**Set**	**Results**	**Search query**
#1	80690	TS = (“college student*” OR “university student*” OR “undergraduate*” OR “undergraduate student*”) AND LA = (English) AND DT = (Article OR Review) AND PY=(2012 to 2021)
#2	98844	TS = (“sleep”) AND LA = (English) AND DT = (Article OR Review) AND PY = (2012–2021)
#3	1841	#1 AND #2

# Represents a prefix for serial numbers.

### Analysis tool

CiteSpace5.8.R3 was created by Dr. Chao Mei Chen, a professor at Drexel University in Philadelphia, the United States, and is an application for data analysis and visualization (27). It has three core concepts, including burst detection, betweenness centrality, and heterogeneous networks, which help identify the nature of research frontiers, label keywords, and detect emerging trends and sudden changes in time (28). The analyzing procedures contain time slicing, thresholding, modeling, pruning, merging, and mapping (27).

The downloaded plain text files were named “download_xx” and then imported into CiteSpace5.8.R3 for format conversion and elimination of duplicate documents. Microsoft Excel 2010 (Redmond, Washington, USA) was used to create line charts according to the annual distribution of the number of published studies. We set the analysis period from 2012 to 2021, set a time slice of 1 year, and used the selection criteria of the G-index. To generate an intuitive and concise map, different pruning methods were set. Through CiteSpace5.8.R3, we analyzed the cooperation networks of authors, countries and institutions, the cited journals, co-cited references, co-occurring keywords, cluster analysis, the citation burst of keywords, and the timeline view of college students' sleep. Several maps of items were constructed. The maps were made up of many nodes and links. The nodes represent the analysis objects, such as authors, countries, or keywords, their size means, the frequency or the number of publications and their colors correspond to the first appearing times. Similarly, links represent the relationships of co-operation or co-citation and their colors show the year they first appeared (29). The degree of connection is reflected by links' thicknesses. Betweenness centrality measures the percentage of the number of shortest paths in a network to which a given node belongs and quantifies the important position of a node (28). A node of high betweenness centrality is usually one that connects two or more large groups of nodes with the node itself in between and is highlighted with high purple trims (30). The thickness of the purple trim reflects the strength of centrality. We considered nodes with high centrality as hot spots or turning points in a field (23).

## Results and analysis

### Annual publications

**Figure 1:**
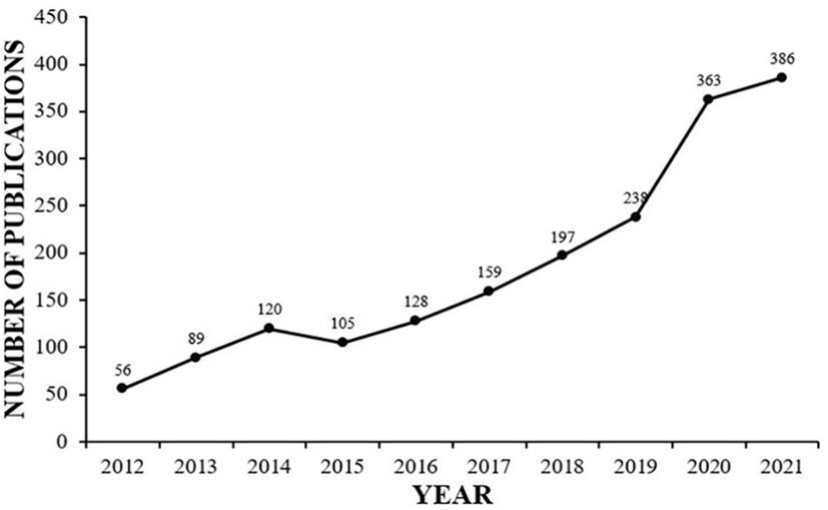
Annual trend chart of publication volume (2012–2021).

A total of 1,841 records were included. We drew a line chart with the year as the abscissa and the number of publications as the ordinate, which is shown in Figure 1. The publications on college students' sleep can be described as having occurred in three stages. In the first stage, from 2012 to 2013, the number of publications was below 100, which meant college students' sleep caught less attention. The second stage was from 2014 to 2019. Even though the line had fluctuations, it steadily increased, and the number of articles published rose from 120 in 2014 to 238 in 2019. Since 2020, the number of articles published on the topic has exploded. The annual number of articles published exceeded 300. The line charts show that this domain continued to develop and has caught greater attention in the past 10 years.

### Co-author analysis

**Figure 2:**
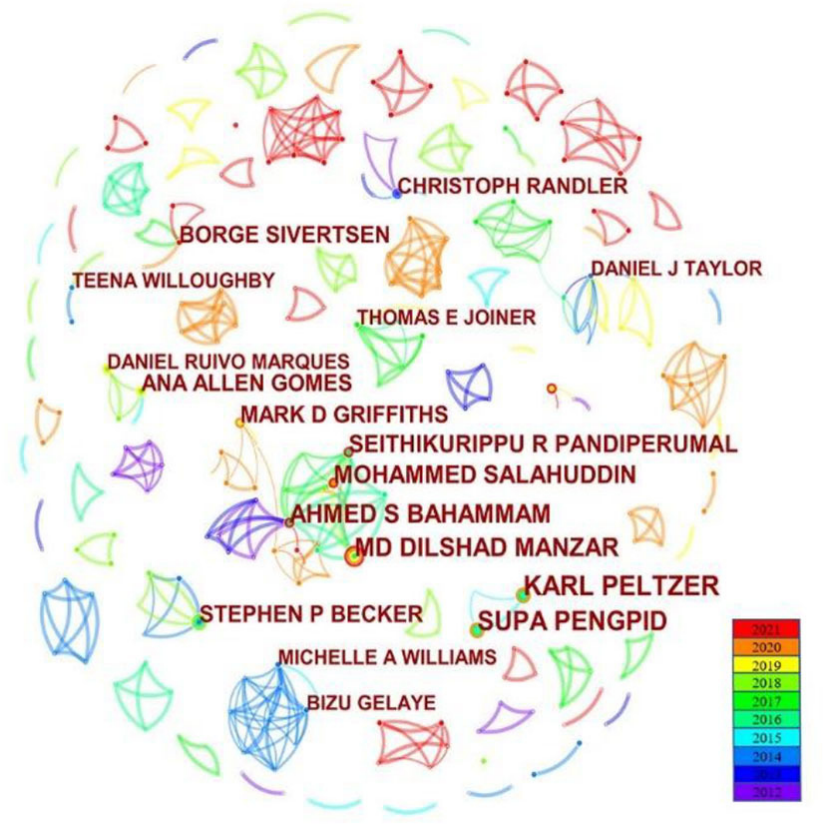
The network map of co-authors.

Using Citespace5.8.R3 to analyze author cooperation networks, we found the authors with high publication volume, cooperative groups among them, and identified the authors with certain influence in this field. As can be seen from Figure 2, there were 398 nodes, 442 links, and some cooperation groups. A larger node size represented a larger number of publications. The line indicated that authors at both ends had a cooperative relationship and its thickness showed us the strength of the cooperation. Colors from cool to warm reflected the time from 2012 to 2021. Karl Peltzer ranked first in terms of publications. He published 15 articles, accounting for 0.8% of the total publications. Supa Pengpid was the second one with 14 articles. They studied the prevalence and related factors of health risks among college students from 26 countries, which made people know more about adolescents' health. Their highest citation article described the current state of sleep duration among college students in 26 countries and found it was associated with sociodemographic variables, health risk behaviors, and health status variables (31). The other top 10 authors' publications ranged from 9 to 13. Although some stable cooperative networks have been formed among the top 10 authors, such as the network between Karl Peltzer and Supa Pengpid. Md Dilshad Manzar, Ahmed S Bahammam, Mohammed Salahuddin, and Seithikurippu R Pandiperumal also had close cooperation with each other. Cross-country cooperation is still relatively low. We are looking forward to promoting the exchange of scholars from different countries. Apart from that, the centrality of all authors was zero, indicating that no authoritative or influential scholar has emerged in the field of college students' sleep.

### Country and institution analysis

**Figure 3:**
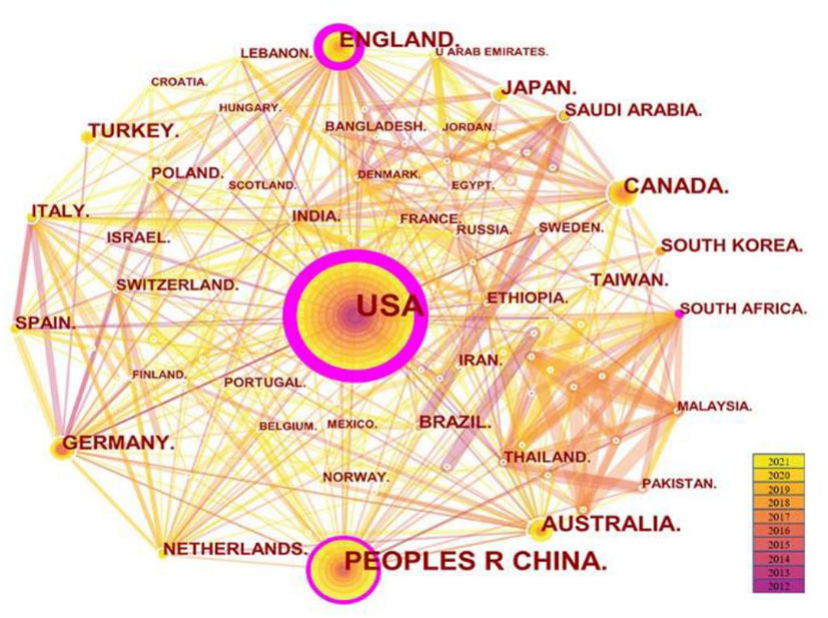
The network map of countries.

Citespace5.8.R3 was used to analyze the cooperation network of countries and institutions. The results are shown in Figure 3 and Table 2. In Figure 3, the map consisted of 100 nodes and 447 links. The node size of the United States was the biggest, with the thickest purple trim which meant highest centrality. The top five countries contributed a total of 1,212 (65.8%) publications, which were the United States with 680 articles (36.9%), China with 250 articles (13.6%), Canada with 95 articles (5.2%), the United Kingdom with 94 articles (5.1%), and Australia with 93 articles (5.1%). Each country has formed a close and stable cooperation network. In terms of centrality, the top five countries were the United States (0.42), the United Kingdom (0.25), China (0.16), South Africa (0.13), and Australia (0.10), which indicated that the United States has made greater contributions than other countries and that developed countries have had a greater impact in the field of college students' sleep.

**Table 2:** Top five institutions in the list by publication volume/centrality.

**Rank**	**Ranked by publication volume**		**Ranked by centrality**	
	**Institution**	**Number**	**Institution**	**Centrality**
1	Harvard University	40	Harvard University	0.23
2	King Saud University	25	Monash University	0.16
3	Brown University	25	The Hong Kong Polytechnic University	0.1
4	University of Pittsburgh	19	Brown University	0.08
5	The University of Alabama	17	University of Calif Irvine	0.07

The top five institutions are shown in Table 2, which were all universities. In terms of volume, the top five institutions are Harvard University (forty articles, 2.17%), King Saud University (twenty-five articles, 1.36%), Brown University (twenty-five articles, 1.36%), University of Pittsburgh (nineteen articles, 1.03%), and The University of Alabama (seventeen articles, 0.92%). Harvard University contributed a lot in this area as a result that it ranked first both in publication volume and centrality. However, only the top 3 institutions' centrality was >0.1, which meant that a majority of institutions played a relatively small role in linking the whole network.

### Analysis of cited journals

We applied CiteSpace5.8.R3 to perform a co-cited analysis of journals, which reflected the relevance of various journals and disciplines. Table 3 lists the top five journals ranked by citation frequency and centrality. Interestingly, they were totally different. The American journal SLEEP had the highest citation frequency with a total of 907 citations, followed by PSYCHIATRY RESEARCH (723) and SLEEP MEDICINE (695). The journal with the highest centrality was INTERNATIONAL JOURNAL OF ENVIRONMENTAL RESEARCH AND PUBLIC HEALTH (0.05), and JOURNAL OF AFFECTIVE DISORDERS (0.05), and the remaining three journals were tied for third place with a centrality of 0.04. The results showed that the literature was always published in journals about sleep, psychology, or psychiatry and the number of publications on college students' sleep was high but scattered. SLEEP was the most frequently cited journal with an impact factor of 5.849 in 2021, and it is in Journal Citation Reports Region I. SLEEP was sponsored jointly by the Association for the Psychophysiological Study of Sleep, the European Society for Sleep Research, and the Association of Sleep Disorders Centers. It includes studies related to sleep and has great influence in the field.

**Table 3:** Top five journals in the list by citation frequency/centrality.

**Rank**	**Ranked by cited frequency**		**Ranked by centrality**	
	**Journal**	**Number**	**Institution**	**Centrality**
1	SLEEP	907	INT J ENV RES PUB HE	0.05
2	PSYCHIAT RES	723	J AFFECT DISORDERS	0.05
3	SLEEP MED	695	BEHAV RES THER	0.04
4	PLOS ONE	638	MED SCI SPORT EXER	0.04
5	SLEEP MED REV	629	PLOS ONE	0.04

### Analysis of cited references

We apply CiteSpace5.8.R3 to conduct a cited references analysis. Table 4 shows the top 10 co-cited references by frequency. The first was the document written by Hannah G. Lund and published on ADOLESCENT HEALTH, which explored college students' current situation regarding sleep disorders, sleep patterns, and predictive factors of sleep quality (32). The cited frequencies were 69 times. The second one was published by Kadir Demirci, which studied the relationship between mobile phone use severity with sleep quality, depression, and anxiety in university students (33). The third co-cited reference focused on the prevalence of sleep disorders, sex differences it existed, and correlations with mental health in a large, multi-university sample of college students (4). Table 5 presents the top 10 studies by centrality. The first reference had a centrality of 0.18 and it was also the second co-cited frequency literature, indicating that the article has the most influence in the domain of college students' sleep (33). It reminded us the abuse of mobile phone would cause s series of problems, especially in sleep. The second reference written by Péter Simor described if the sleep problems impacted the association between chronotype and negative emotions, and if eveningness preference led to bad emotions (34). The third reference found that good global sleep quality could reduce the happening of drinking-consequences, which confirmed the importance to maintain a healthy lifestyle (35). Browsing these cited references listed in Tables 4, 5, we were able to find that most research focused on the characteristics of sleep, its association with health- related behaviors and psychological problems, and the outcomes of sleep disorders. Except that, high-quality guidelines and reviews also appeared.

**Table 4:** Top 10 studies in the list by co-citation frequency.

**Rank**	**Author**	**Co-cited reference**	**Frequency**
1	Lund HG	Sleep patterns and predictors of disturbed sleep in a large population of college students	69
2	Demirci K	Relationship of smartphone use severity with sleep quality, depression, and anxiety in university students	49
3	Becker SP	Sleep in a large, multi-university sample of college students: sleep problem prevalence, sex differences, and mental health correlates	44
4	American Psychiatric Association	Diagnostic and statistical manual of mental disorders DSM-5 5th.	41
5	Hirshkowitz M	National Sleep Foundation's sleep time duration recommendations: methodology and results summary	39
6	Adan A	Circadian typology: a comprehensive review	38
7	Hershner SD	Causes and consequences of sleepiness among college students	36
8	Gaultney JF	The prevalence of sleep disorders in college students: impact on academic performance	32
9	Cao WJ	The psychological impact of the COVID-19 epidemic on college students in China	31
10	Taylor DJ	Epidemiology of insomnia in college students: relationship with mental health, quality of life, and substance use difficulties	30

**Table 5:** Top 10 studies in the list by co-citation centrality.

**Rank**	**Author**	**Co-cited reference**	**Centrality**
1	Demirci K	Relationship of smartphone use severity with sleep quality, depression, and anxiety in university students	0.18
2	Simor P	The influence of sleep complaints on the association between chronotype and negative emotionality in young adults	0.13
3	Kenney SR	Global sleep quality as a moderator of alcohol consumption and consequences in college students	0.12
4	Van Reen E	Current alcohol use is associated with sleep patterns in first-year college students	0.1
5	Adan A	Circadian typology: a comprehensive review	0.09
6	Almojali AI	The prevalence and association of stress with sleep quality among medical students	0.09
7	Urban R	Morningness-eveningness, chronotypes and health-impairing behaviors in adolescents	0.09
8	Lemma S	Sleep quality and its psychological correlates among university students in Ethiopia: a cross-sectional study	0.09
9	Hirshkowitz M	National Sleep Foundation's sleep time duration recommendations: methodology and results summary	0.08
10	Besoluk S	Morningness-eveningness preferences and academic achievement of university students	0.08

### Analysis of co-occurring keywords

**Figure 4:**
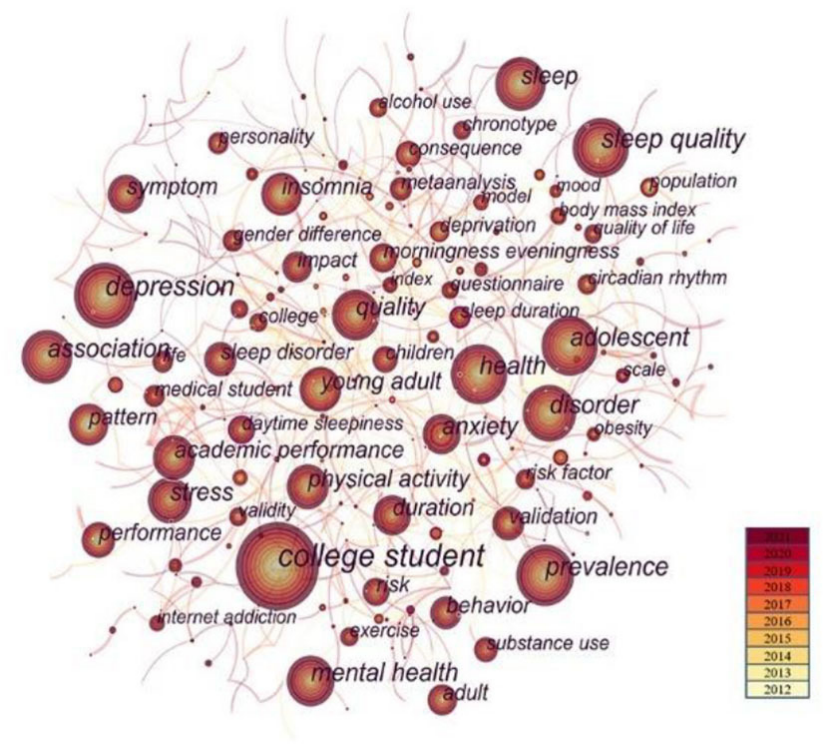
Co-occurring keywords map.

We used CiteSpace5.8.R3 to perform co-occurring keyword analysis. In order to make the map more vivid, we decided to prune the map. We generated a network map of keywords consisting of 499 notes and 785 links, which can be seen in Figure 4 and Table 6. The larger the node's size was, the more frequently the keyword occurred. The yellow color indicated earlier occurrence and the red color meant it appeared in recent years. Keyword co-occurrence refers to the frequency of two topic terms appearing in the same article and is aimed at illustrating the connection between the two topics. Keywords are the core summary of an article, and high-frequency keywords can represent the hot topics. High-centrality keywords indicate that it has a high status and great impact on the field. The hot keywords with high frequency were college student (frequency: 791), depression (frequency: 355), and sleep quality (frequency: 305). The top three high-centrality keywords were adolescence (centrality: 0.18), high school (centrality: 0.17), and transition (centrality: 0.17). It could be concluded from the keywords that college students suffered from a lot of changes in their life and the current situation, characteristics of sleep, and its association with students' mental health and alcohol drinking caught the researchers' attention. Different scales were applied to measure the health problems more normatively.

**Table 6:** Top 10 keywords in the list by co-citation frequency/centrality.

**Rank**	**Ranked by co-citation frequency**		**Ranked by centrality**	
	**Keyword**	**Frequency**	**Keyword**	**Centrality**
1	College student	791	Adolescence	0.18
2	Depression	355	High school	0.17
3	Sleep quality	305	Transition	0.17
4	Prevalence	255	Scale	0.16
5	Association	242	Inventory	0.15
6	Mental health	232	Alcohol	0.14
7	Disorder	225	Individual difference	0.13
8	Health	211	Executive function	0.13
9	Adolescent	211	Daytime sleepiness	0.12
10	Sleep	202	Sleep duration	0.12

### Analysis of keyword clusters

**Figure 5:**
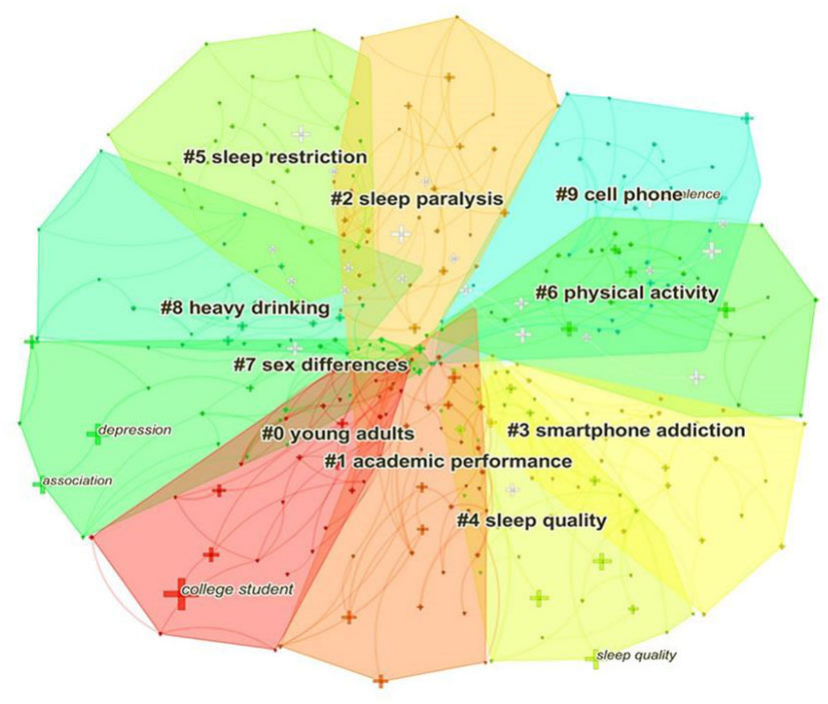
Keyword cluster map. # Represents a prefix of the serial number.

Based on co-occurring keywords, we used log-likelihood tests to generate the map of keyword clusters. Cluster analysis divides keywords into relatively homogeneous modules based on their similarity, dissimilarity, and affinity to each other. The cluster label is extracted from the title, abstract, or keyword. Modularity Q and Silhouette are two important indicators to evaluate the clusters. The Modularity Q represents the modularity of the network, and its value ranges from 0 to 1. A larger value indicates a better clustering result, which reflects a clear identification of sub-domains. Q > 0.3 means that the network is important (30). Silhouette represents the efficiency of clusters. It is a measure of network homogeneity and ranges from 0 to 1, too. The larger the value is, the closer the connection between nodes in the cluster is. S > 0.7 means that the cluster is convincing and S > 0.5 represents that the cluster is reasonable (36). In our study, 21 clusters were generated, and the top 10 clusters labeled with different colors are shown in Figure 5. The Q value was 0.7543 and the Silhouette value was 0.8142, suggesting the cluster results were meaningful and convincing. The top three clusters were young adults, academic performance, and sleep paralysis. Academic performance was the biggest cluster next to the young adults, showing that lots of publications illustrated the connection between sleep and academic performance. Sleep could affect students' academic performance, which was a critical indicator of their competence and intelligence. To some extent, the grade point average could be predicted by sleep duration (37), sleep quality (38), and the frequency and consistency of nighttime sleep awakening (39). In total, the top 10 cluster results expressed that most studies were committed to describing the connections between sleep problems with academic performance, substance addiction, and physical activity. Table 7 lists the details of each cluster.

**Table 7:** Keyword clusters and their keywords.

**Cluster**	**Silhouette**	**Label**	**Keywords**
0	0.901	Young adults	Young adults; sleep quality; college student; symptom; cognitive behavioral therapy; inventory;
1	0.854	Academic performance	Academic performance; biological sex; sleep status; sleep health; chronotype;
2	0.829	Sleep paralysis	Sleep paralysis; sleep quality; sleep duration; creative thinking; sleep timing; young women;
3	0.944	Smartphone addiction	Smartphone addiction; problematic smartphone use; college; delayed sleep-wake phase disorder; sleep disturbance; social cognition;
4	0.942	Sleep quality	Sleep quality; college students; muscle strength; circadian rhythm; class schedule; munich chronotype questionnaire;
5	0.805	Sleep restriction	Sleep restriction; sleep quality; conditional process model; negative emotions; cognitive performance; task difficulty
6	0.977	Physical activity	Physical activity; sleep quality; screen time; risk assessment; big data management; parental myopia
7	0.894	Sex differences	Sex differences; college students; ecological momentary assessment; daytime sleepiness; young adults; family functioning
8	0.726	Heavy drinking	Heavy drinking; mental health; university students; secondhand harm; hangover; prevention
9	0.844	Cell phone	Cell phone; sleep quality; sleep duration; mediation analysis; dietary intake; young women

### Analysis of timeline view

**Figure 6:**
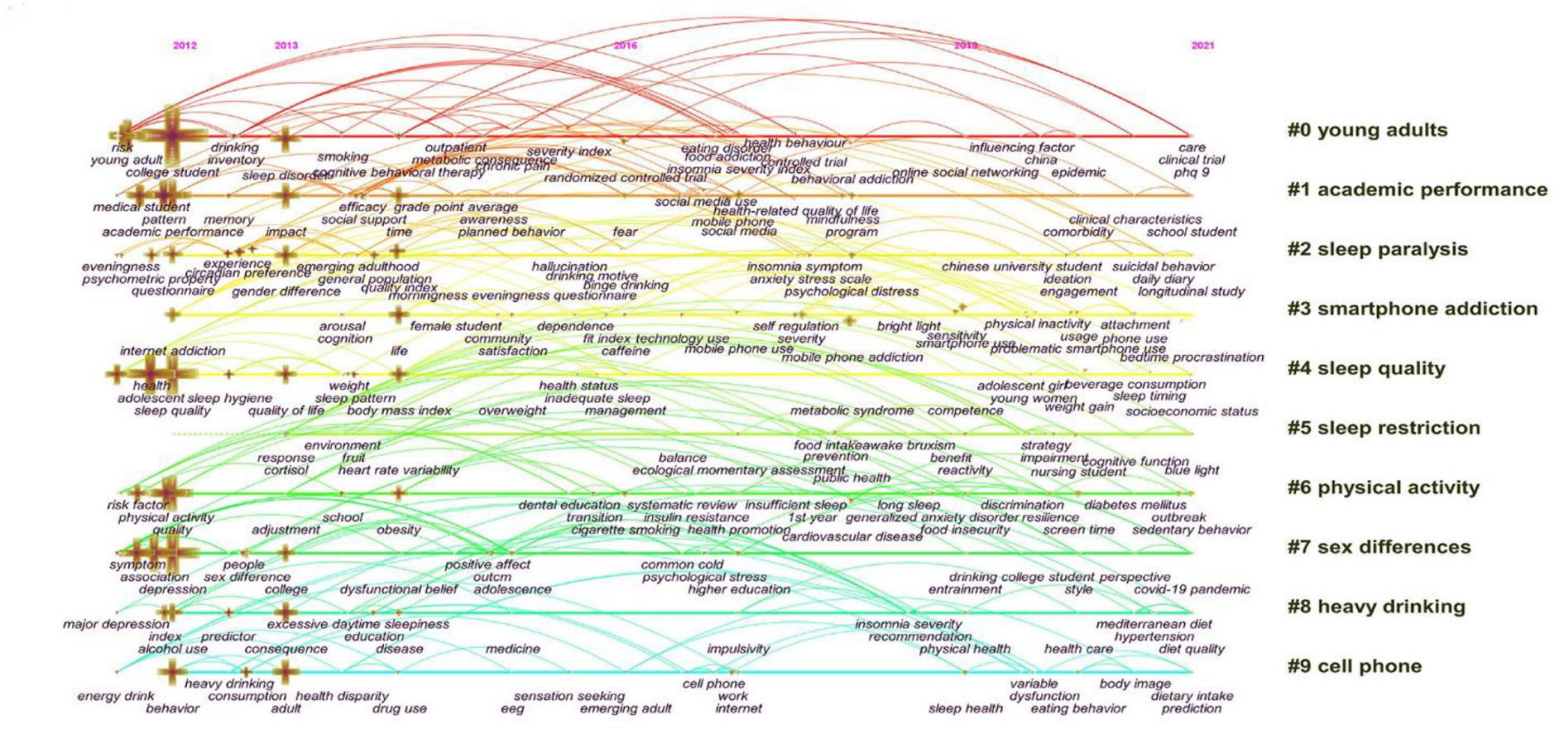
Timeline view map of keywords. # Represents a prefix for serial numbers.

We used CiteSpace5.8.R3 to generate the timeline view analysis. Timeline view can show the relationship of clusters and the evolution span of clusters' keywords over time. Different colors represent different clusters. As shown in Figure 6, overall, the number of keywords has increased gradually over the last decade, indicating that university students' sleep is a meaningful research question among scholars. Clusters 0, 1, and 2 have the highest number of keywords in the decade, indicating that scholars were concerned about sleep problems among university students and their relationships with academic performance. The keywords of #3 smartphone addiction, #5 sleep restriction, and #6 physical activity were initially rare in 2012, but there has been an explosive increase in the number in recent years, suggesting these clusters started to catch scholars' attention. In the timeline view, we could find that there also existed a lot of keywords in 2021, such as sedentary behavior, clinical trial, daily diet, and so on. All of them are the research hot spots in college students' sleep.

### Keywords with citation burst

**Figure 7:**
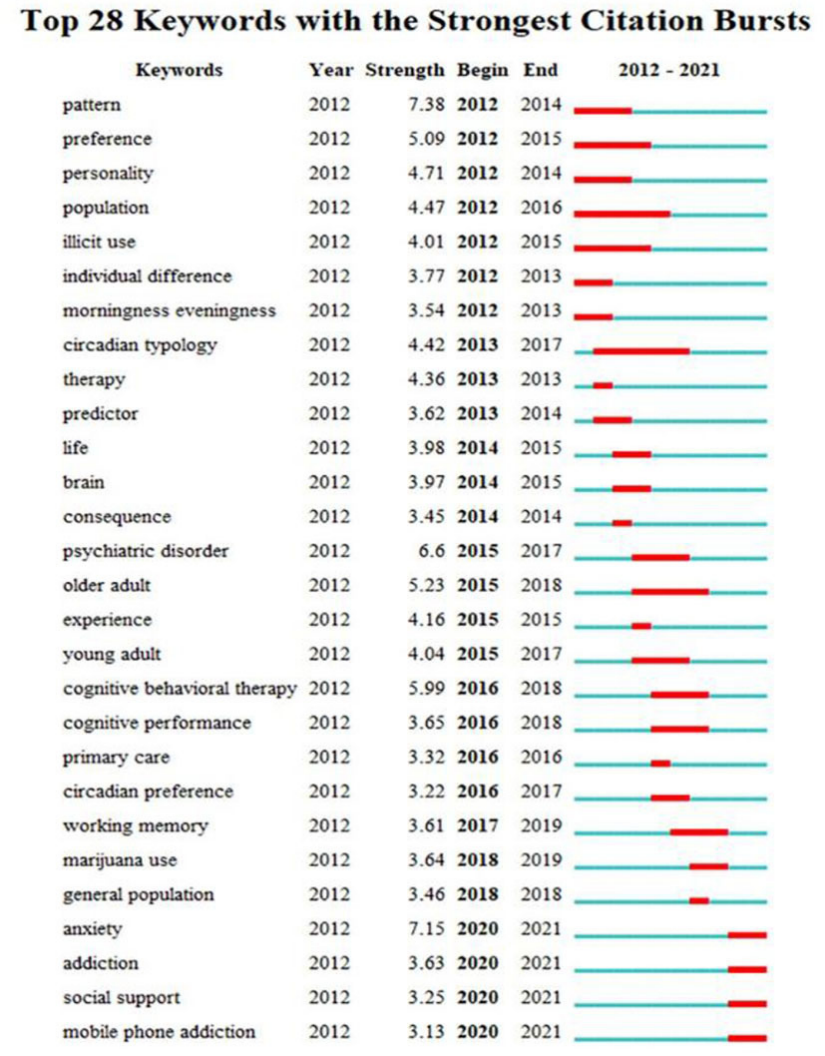
Top 28 keywords with the strongest citation bursts.

We used CiteSpace5.8.R3 to visualize the keywords citation burst. Burst detection depends on Kleinberg's algorithm, which helps extract the meaningful structure from document streams and identify the keywords that experienced abrupt changes (40). The keywords citation burst can show the time distribution and dynamic change trends for keywords at a certain stage, which helps us learn more about past and present hot spots. Figure 7 presents the top 28 strongest citation bursts. The blue line represents the time interval and the red line represents the keywords citation burst (41). The hot spots could be described as three stages. It is known that the research focused on college students' sleep patterns, circadian typology, and the consequence and predictors of sleep problems from 2012 to 2014. From 2015 to 2018, the association between sleep with psychiatric disorders or cognitive functions, and the effectiveness of cognitive behavioral therapy became hot spots in this period. The latest research hot spots are shown from 2020 to 2021. Anxiety, social support, and substance addiction are becoming popular topics in college students' sleep now.

## Discussion

The current study used CiteSpace5.8.R3 to conduct a bibliometric analysis of literature in the field of college students' sleep over the past 10 years on the Web of Science, and maps and tables helped display the research status, hot spots, and frontiers more intuitively and clearly. The number of publications in this field has shown a gradual increase over the past decade, indicating that researchers have made more efforts to publish articles about college students' sleep. The USA, China, and Canada, as well as universities and medical institutions have researched the field most intensively and contributed the most articles and influences. Karl Peltzer was the most prolific author with 15 articles and engaged in close collaboration with Supa Pengpid. No author with high centrality has appeared yet, which means that the degree of collaboration and influence of the authors is not sufficient. The existing articles are mainly published in journals related to sleep, psychiatry, or psychology. The most frequently cited journal is SLEEP, a journal that mainly includes research about sleep. The hot spots are associations between sleep problems with physical and mental health and substance use disorders. What's more, research about population differences in sleep are also growing now. Future studies will be devoted to conducting clinical trials to find out effective interventions for sleep disorders. In addition, the complex connections between sleep and anxiety, smartphone addiction, and other health-related behaviors will be studied further through more rigorous studies.

### Cooperation is necessary

Although many countries and authors have devoted a lot to this field, the distribution of research contributions is very uneven. Except for China, other productive countries are all developed countries. Most of the top five institutions come from the USA. Developing countries seem to occupy a small part of publications in this field. We hope that developing countries will conduct more research and cooperate more with other countries or institutions. Next, in the network map of co-authors, cooperative networks were formed with different densities between authors, but most of the networks were limited to small groups. Although Ahmed S Bahammam was not the most prolific author, he connected three cooperation networks and the cooperation helped authors in them produced more articles, which implied the benefits and importance of cooperation. As a result, it is necessary to expand the authors' connections to conduct research on sleep more in-depth and produce high-quality literature in the future. In this way, the research literature will become more globally applicable.

### The research hot spots

Keywords are highly concise summaries of literature, and clusters can classify keywords according to algorithms. We combined keywords with cluster analysis and summarized the three research hot spots in this field.

#### Prevalence of sleep disorders and individual differences

Sleep is an important physiological function of the human body. Good sleep can eliminate fatigue and restore vitality. Healthy sleep plays an important role in maintaining the brain's function and cognition (42). Common sleep disorders include long sleep latency, insomnia, early awakening, and a sleep duration that is too short or too long. In the past 10 years, the research about the prevalence of sleep disorders among college students have grown continuously. The prevalence of poor sleep quality among undergraduates ranged from 9.8% to 63.39% (11, 43, 44). Interestingly, when we compare the earlier literature with recent literature, we can find some differences between them. The earlier literature tended to investigate ordinary college students' sleep (45). The latest literature, however, tended to focus on sleep disorders among different groups, such as different genders or races (46, 47). As the research progresses, more research about the prevalence of sleep problems among different groups of college students will be conducted. As a result, we can have a more accurate understanding of sleep problems in different populations, according to which future targeted interventions will also be designed for various populations.

#### The associations between sleep and physical and mental health

In the top 10 co-citation frequency keywords, depression and mental health were included. In addition, cluster 6 physical activity with the highest silhouette value of 0.977 also contained more keywords with time. More publications surveyed the relationships between sleep and physical and mental health. They influenced each other through complex and bilateral interactions.

Mental problems contain negative emotions such as depression, anxiety, stress, and so on. College students experienced many changes in university and were more likely to suffer from mental problems coming from the study, social communication, and work. A study that included students from six universities showed that poor sleep quality was linked to depression and anxiety (4). A systematic review combined 34 studies found that poor sleep quality and insomnia were associated with stress (48). However, most of the studies were cross-sectional and could not prove a causal relationship between them. Recently, some longitudinal studies confirmed that there was a bidirectionally predictive relationship between poor sleep quality and mental health problems (49, 50). But this is not enough and more studies are needed to supplement and support current findings. In the future longitudinal studies in a large sample should be done to verify the complex mechanism between sleep and mental health.

University students reported having less physical activity, which led to poor health outcomes (51, 52). A lot of publications described the association between sleep and physical activity. A literature review showed that physical activity significantly altered sleep quality (53). It raised that interventions in physical activity may improve sleep. Memon et al. included 29 literature in a systematic review and found that moderate-to-high intensity physical activity was concerned with better sleep quality in university students, but there existed differences in various counties and sleep indicators (54). Cross-sectional and longitudinal evidence on the association between them is still equivocal. Researchers should consider sleep and physical activity as codependent behaviors and conduct high-quality studies in different populations to enrich the conclusions afterward.

#### The relationships between sleep and problematic substance use behaviors

In keywords clusters and timeline view, we can classify smartphone addiction, heavy drinking, smoking, and food addiction as problematic substance use behaviors. Tayana Panova summarized the theoretical definition of addiction for two key points: the severe harm or negative consequences and the psychological and physical dependence that leads one to carry on the behavior (55).

The prevalence of problematic substance use behaviors in college students was high and led to a range of psychological problems (56). Recent studies proved that smartphone and other electronic devices became risk factors for sleep quality (57, 58). Sleep disorders also increased the risk of alcohol use disorder and alcohol consumption contributed to bad sleep quality in adolescents (59, 60), which may depend on alteration of brain structure and neuropsychological mechanism (61). Considering that problematic substance use behaviors harmed college students' sleep health, more scholars conducted studies to find the pathways of influences between them (62, 63). Path analysis provides changeable directions, allowing for better and more feasible interventions. For example, interventions targeting problematic smartphone use and psychological mood may provide a buffer for the negative impact of poor sleep quality on eating disorder symptoms through mediating effects (64). With the in-depth research, more indirect relationships will be explored to extend the current discovery. If possible, scholars or clinical specialists can apply related theories to make interventions on mediating or moderating factors.

The Theory of Planned Behavior is highly predictive of human functioning and it contains three latent variables: perceived behavioral control, subjective norms, and attitude toward the behavior (65, 66). A study found that perceived behavioral control was a strong predictor of both behavioral intention and sleep behavior (67). Health Belief Model constructs were significantly associated with poor sleep quality, too (68). We are looking forward to credible research results which will change the problematic behaviors and improve the students' sleep health.

### The development trends and frontiers in college students' sleep

#### The development trends of hot spots

By analyzing the timeline view and keywords citation burst, we divided the research hot spots of college students' sleep into three stages.

From 2012 to 2014, the study of college students' sleep was at the beginning stage. According to the keywords of pattern, preference, personality, circadian typology in keywords citation burst and association, risk, and impact in timeline view, it is obvious that the studies conducted at this time were devoted to illustrating the status quo, affecting factors, and consequence of college students' sleep problems from a broad angle (69). It was reasonable as a result that the scholars were unclear about these research subjects and they needed to have a general impression of college students' sleep quickly. Chronotype was a hot topic in this stage, particularly concerning students. Chronotype represents people's circadian rhythmicity, which contains three types: morning-types, evening-types, and neither-types (70). It reflects students' sleep preference and more or less impacts their health or life (71, 72).

The second stage was from 2015 to 2017, the associations between health-related behaviors and psychological disorders with sleep were explored further, while guidelines, specific scales, intervention studies, and systematic reviews began to emerge. Based on research in stage one, studies on related factors became more concrete, and scholars investigated further and tried to clarify the mechanism in them. Additionally, an increasing number of scholars started to detect effective interventions for sleep disorders, most of which were psychological interventions or over-the-count drugs or herbs (73, 74). A systematic review classified the types of psychological interventions as sleep hygiene education, cognitive–behavioral therapy, relaxation, mindfulness, hypnotherapy, and other psychotherapeutic interventions. Among them, cognitive-behavioral therapy was the most effective in improving students' sleep (75). In the future, more interventions should be identified and implemented to verify their effectiveness in addressing sleep problems. Meanwhile, considering the significance of research tools, more research launched on designing and verifying the validity of scales, making the tools more mature (76). Besides, some researchers summarized the existing results and wrote guidelines or systematic reviews for interested scholars or clinical workers to refer to (77, 78).

From the second stage to now, the objects of studies have become more refined, including smartphone addiction, anxiety, sedentary behavior, or daily diet (64, 79). One reason is that researchers have learned more about sleep gradually, and another may be that students' lives changed dramatically during the epidemic, which affected their physical and mental health and health-related behaviors. Research on the diet and sleep of college students seems to become more popular than before. Students who ate unhealthily were more likely to have poor sleep quality (80). Most studies concentrated on the complicated connections between two and more variables in a specific population or background. Longitudinal studies are advised and done to confirm their assumptions to know more about the casual relationship (50, 81). It is interesting to screen the development of research hot spots and see the constant deepening of the studies in this field.

#### The research frontiers

In the keywords burst analysis, the keywords with the strongest citation burst are particularly suitable for spotting new trends and sudden changes. Anxiety, addiction, and social support have the strongest burst strength above 3, which can be identified as the frontiers. It is also in line with current research hot spots in the third stage. We sum up three research trends in the future: firstly, the complex relationships between sleep and substance addiction, mental health, and diet quality should be supplemented, such as mediating or moderating effects; secondly, scholars ought to conduct longitudinal studies or randomized controlled trials to confirm the affecting factors or adverse outcomes of sleep and find useful interventions on sleep problems, especially in diverse populations; finally, objective assessment tools like polysomnography or actigraph are expected to be applied in more research, adding the reliability of research results.

### Strengths and limitations

The highlights of the study are that it is the first bibliometric analysis of the literature on college students' sleep from 2012 to 2021, conducted *via* CiteSpace5.8.R3. We used several vivid knowledge maps and tables to show the developing process, research hot spots, and frontiers of college students' sleep. Furthermore, high-quality literature from the Web of Science Core Collection Database was chosen and the analysis results have some reference value, which can, thus, provide guidance for further research. There are also limitations of this paper. We only included the English literature retrieved by a specific search string from one database published in the last decade. Therefore, we could not include all the literature and some less relevant literature may be analyzed, which may affect the generalization of the results. If possible, prospective bibliometric analysis in this field should design a more perfect retrieve string to include more literature with no restrictions on languages, document types, or publication times.

## Conclusions

In conclusion, the current study provides a visualization analysis of the relevant literature in the field of college students' sleep and clarifies the related research hotspots and frontiers. We conclude that research hot spots are the prevalence of sleep disorders in different populations and the complicated relationship between sleep and physical and mental health and substance use disorders. Longitudinal studies and randomized controlled trials are necessary to expand the current results. Our research served as a guidance and reference for researchers to think and determine their research direction, reducing the efforts to explore the boundaries of the field. Educators and clinical workers can carry out sleep hygiene interventions and practices in a targeted way to improve the sleep health of students. In addition, collaborations between countries, institutions, and authors need to be strengthened to conduct more in-depth research. All of the research purposes are to maintain the global college student's health.

## Data availability statement

The raw data supporting the conclusions of this article will be made available by the authors, without undue reservation.

## Author contributions

JiQ and HS had contributions to the study design. YH and YB searched the database and collected the data. JZ, SJ, and JuQ together analyzed the data. JZ, TZ, and MX were responsible for drafting the manuscript and interpreting the results. YL gave professional advice and edited the manuscript. All the authors read and approved of the final manuscript.

## Funding

This work was supported by Aging Education Learning Resource Library Sub-library Project in Jiangsu Province in 2019.

## Conflict of interest

The authors declare that the research was conducted in the absence of any commercial or financial relationships that could be construed as a potential conflict of interest.

## Publisher's note

All claims expressed in this article are solely those of the authors and do not necessarily represent those of their affiliated organizations, or those of the publisher, the editors and the reviewers. Any product that may be evaluated in this article, or claim that may be made by its manufacturer, is not guaranteed or endorsed by the publisher.
